# Radio Frequency Detection and Characterization of Water-Ethanol Solution through Spiral-Coupled Passive Micro-Resonator Sensor

**DOI:** 10.3390/s18041075

**Published:** 2018-04-03

**Authors:** Gyan Raj Koirala, Rajendra Dhakal, Eun-Seong Kim, Zhao Yao, Nam-Young Kim

**Affiliations:** 1 RFIC Lab, Department of Electronic Engineering, Kwangwoon University, 01897 Seoul, Korea; thegrkoirala@gmail.com (G.R.K.); aperioraj@gmail.com (R.D.); 3037eskim@gmail.com (E.-S.K.); yao9074@hotmail.com (Z.Y.); 2 Department of Computer Science and Engineering, Sejong University, 05006 Seoul, Korea

**Keywords:** micro-resonator sensor, permittivity, radio frequency, reproducible, water-ethanol

## Abstract

We present a microfabricated spiral-coupled passive resonator sensor realized through integrated passive device (IPD) technology for the sensitive detection and characterization of water-ethanol solutions. In order to validate the performance of the proposed device, we explicitly measured and analyzed the radio frequency (RF) characteristics of various water-ethanol solution compositions. The measured results showed a drift in the resonance frequency from 1.16 GHz for deionized (DI) water to 1.68 GHz for the solution containing 50% ethanol, whereas the rejection level given by the reflection coefficient decreased from −29.74 dB to −14.81 dB. The obtained limit of detection was 3.82% volume composition of ethanol in solution. The derived loaded capacitance was 21.76 pF for DI water, which gradually decreased to 8.70 pF for the 50% ethanol solution, and the corresponding relative permittivity of the solution decreased from 80.14 to 47.79. The dissipation factor increased with the concentration of ethanol in the solution. We demonstrated the reproducibility of the proposed sensor through iterative measures of the samples and the study of surface morphology. Successive measurement of different samples had no overlapping and had very minimum bias between RF characteristics for each measured sample. The surface profile for bare sensors was retained after the sample test, resulting a root mean square (RMS) value of 11.416 nm as compared to 10.902 nm for the bare test. The proposed sensor was shown to be a viable alternative to existing sensors for highly sensitive water-ethanol concentration detection.

## 1. Introduction

Radio frequency (RF) and microwave sensors have been widely studied as viable approaches for label-free detection of polar (bio)molecular solutions, including glucose [1,2,3], deoxyribonucleic acid (DNA) [4], proteins [5], and hydroxyl group compounds [6,7]. The frequency-dependent response of these solutions during their interaction with time-varying electromagnetic fields allows for cost-effective, mediator-free detection of (bio)molecules within a very short assay time, thereby establishing a distinctive route to electrochemical [8] and optical sensing [9,10]. Advancements in microfabrication technology have further enabled the realization of bio-responsive, high-performance, compact microelectronic devices compatible with lab-on-a-chip technology [6,11].

Understanding how the dielectric properties of aqueous solutions are governed by their permittivity characteristics is fundamental to their electromagnetic detection. Various methodologies, such as (complementary) split-ring resonators [5,12,13] and interdigital capacitors [14,15], have been adopted for the characterization of biomolecules in the microwave region. In our previous work [16], we developed an air-bridge capacitor-based microsensor realized through integrated passive device (IPD) technology for the effective characterization of the complex permittivity of human serum.

The extensive use of ethanol in industry for chemical, pharmaceutical, and food and beverage applications has increased the need for regular monitoring of the water-ethanol composition to ensure product quality. In this regard, several microwave techniques have been proposed for the sensing and characterization of water-ethanol mixtures. The real-time sensing of water-ethanol and glucose mixtures using multifrequency operable planar microstrip bandpass filter (BPF) has been suggested with a chamber flow-through path [17]. Chretiennot et al. have proposed the use of a microfluidic planar resonator sensor for complex permittivity characterization of water-ethanol solutions [6]. However, such sensors require that a high sample volume pass through the microcavity for sensitive detection. In addition, the operating frequency of the microfluidic planar resonator sensor is very high (>15 GHz), making implementation in a modern communication system that predominantly operates at L- and S-bands difficult. A sensor based on a complementary planar split-ring resonator, which operates at 2.4 GHz, has been proposed for microfluidic dielectric characterization of water-ethanol solutions [18]; however, the size of the device is relatively large, and the copper conducting layer undergoes resistive loss and suffers from oxidation in an open environment.

In this study, we propose a microfabricated resonator that incorporates passive components for selective and reproducible detection of varied volume-to-volume concentrations of water-ethanol solution. The complex permittivity analysis of different water-ethanol solution concentrations was determined to ensure the resonance frequency change was noticeable. The results were then compared with the results of the standard Debye relaxation process for water-ethanol solution. The obtained results were precise enough having shown an average standard deviation of 0.8% and 5.15% for real and imaginary permittivity, respectively. The proposed sensor allows the smallest detectable concentration of ethanol (3.82%) from the water-ethanol solution. The antifouling process was analyzed using the surface characterization at various stages and instances of the experiment. The atomic force microscopy (AFM) and RF results revealed that there was no considerable variation in surface morphology that would alter the frequency-dependent electromagnetic properties of the sensing surface [19], maintaining the unique resonance frequency corresponding to the unique concentration of the water-ethanol solution. The concept of implementing passive components in the form of an RF sensor via IPD technology was shown to be a promising method for the development of reproducible and label-free ethanol concentration detection.

## 2. Materials and Methods

### 2.1. Design and Fabrication

**Figure 1:**
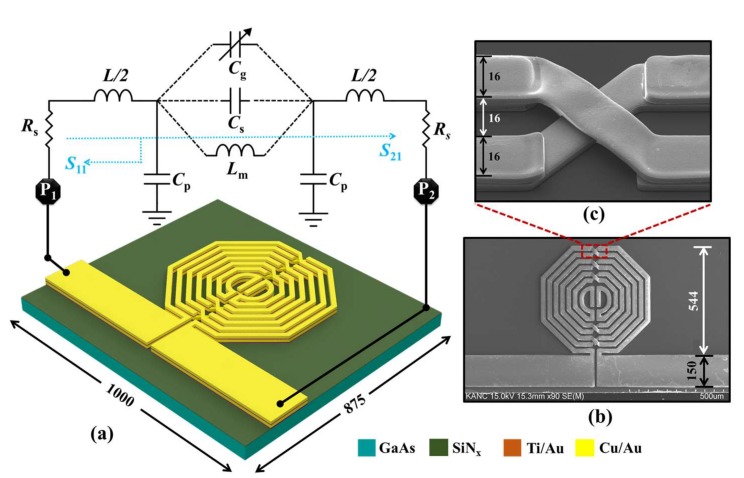
Schematic diagram of the proposed sensor. (**a**) The multilayered spiral-coupled octagonal resonator structure realized through IPD processing has a total size of 1 mm × 0.875 mm, shown with its equivalent lumped-element circuit; (**b**) Scanning electron microscopy (SEM) image of the fabricated sensor; (**c**) An enlarged view of the air-bridge interconnection. (Note: all dimensional units are in micrometers).

A BPF comprising multilayered, spiral-coupled, open-ended, octagonal resonators was designed and simulated using full-wave simulation software, SONNET, as shown in Figure 1. The resonators are terminated with 50-Ω impedance-matching transmission lines. The width of the resonators and the gap between two consecutive resonator lines were both maintained constant at 16 µm. The device was fabricated using IPD technology in a highly resistive gallium arsenide (GaAs) substrate owing to a wide bandgap energy, having a relative dielectric constant of 12.85 and a thickness of 200 µm. The fabrication process is shown in supplementary Figure S1, which includes details on the materials, thicknesses, and process involved in each step. The post-fabrication process involved wire-bonding the device to the printed circuit board (PCB) for safe measurement and handling. The realized IPD-based sensor was shown to be an appropriate candidate for the sensitive detection of the water-ethanol concentration, inheriting the functionality of previously proposed IPD-based glucose sensors such as reusable, robust, and mediator-free operation [3,16,20].

### 2.2. Radio Frequency Modeling

The spiral-coupled BPF is shown in Figure 1a as a lumped-element equivalent-circuit component model and a passive resonator model, incorporating two intertwined symmetrical resonators to form a resonant circuit. The realized BPF utilized mixed coupling comprised of inductive coupling (*L*_m_) between the intertwined resonators, each having an inductance of *L*/2, gap capacitive coupling (*C*_g_) generated between the gaps of the two open-ended intertwined resonators, and residual capacitance (*C*_s_) developed through the substrate. The resistive property of the resonator was modeled by *R*_s_, and the capacitance *C*_p_ represented the capacitance between the metal surface and the ground plane. The introduction of aqueous solution increases C_g_, which in turn decreases the resonance frequency of the resonator as a function of varied dielectric permittivity. Figure 1b shows the scanning electron microscopy (SEM) image of the fabricated device; the cross-sectional view of the two intertwined metal layers, which are connected through an air-bridge underpass, is shown in Figure 1c. The multilayered resonators utilizing the air-bridge connection facilitated the realization of a compact chip while maintaining an acceptable quality factor [21,22].

The fundamental resonance frequency $\left(f_{o}\right)$ of the BPF is given by the following formula:

(1) $$f_{o}=\frac{1}{2\pi \sqrt{L_{eq}C_{eq}}},$$

where *L_eq_* is the equivalent inductance of the octagonal sensor and *C_eq_* is the total capacitance. In (1), the equivalent inductance of the octagonal resonator can be approximated from

(2) $$L_{eq}=K_{1}\mu_{o}\frac{n^{2}d_{avg}}{\left(1+K_{2}\rho \right)},$$

where $K_{1}=2.25$ and $K_{2}=3.35$ are constants, $\mu_{o}=4\pi \times 10^{-7}H/m$ is the absolute permeability, $d_{avg}=\left(d_{out}+d_{in}\right)/2$ is the average of the outer and inner diameter of the octagonal resonator, and $\rho =\left(d_{out}-d_{in}\right)/\left(d_{out}+d_{in}\right)$ is the fill ratio. The unit cell capacitance $\left(C_{uc}\right)$ is the summation of the distributed capacitances

(3) $$C_{uc}=C_{g}+C_{s}.$$

The gap capacitance (*C_g_*), and substrate guided residual capacitance (*C_s_*) can be approximated from the following relationship [23] using an elliptic integral of the first kind K[x] for the transformation:

(4) $$C_{g}+C_{s}=\varepsilon_{o}\left[\frac{\left(\varepsilon_{s}+\varepsilon_{sub}\right)}{2}\frac{K\left[\sqrt{1-{\left(g/(g+w)\right)}^{2}}\right]}{K\left[g/\left(g+w\right)\right]}+\frac{h}{g}\right].$$

Here, $\varepsilon_{sub}$ is the dielectric constant of the substrate, $\varepsilon_{s}$ is dielectric constant of the sample (=1 for bare sensor), w is the width of the resonator, g is the gap between the resonators, and *h* is the height of the metal line. Thus, the total capacitance is $C_{eq}=C_{uc}L$, where *L* is the total length of the resonator.

From the simulation results, the resulting lumped-element parameters of Equation (1) were obtained as *L_eq_* = 0.72 nH and *C_eq_* = 3.2 pF, which generated a resonance frequency at 3.3 GHz with a rejection level of 27.96 dB. There was good agreement between simulated and measured results, which showed a resonance frequency of 3.24 GHz with a rejection level of 33.33 dB (Figure S2).

### 2.3. Sensor Characterization

**Figure 2:**
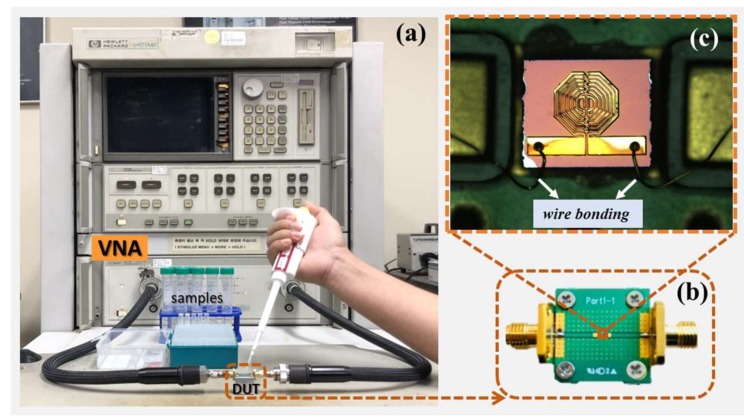
(**a**) Measurement setup including the device-under-test (DUT), test samples, a micropipette for depositing the samples, connection probes, and a network analyzer for examining the performance of the device under varied concentrations of water-ethanol solution. (**b**) Enlarged view of the DUT. (**c**) Microscopic image of the fabricated sensor, which was connected to the planar circuit board through wire bonding.

Seven different volume-to-volume concentration samples of 10 mL each were prepared, taking volume percentages of 5, 10, 20, 30, 40, and 50 of ethanol mixed in DI water. The performance of the sensor was evaluated by measuring the scattering parameters using the Agilent 8510C network analyzer (Agilent Technologies, San Diego, CA, USA). The full two-port short-open-load-through (SOLT) calibration was defined prior to the measurements. Figure 2 shows the measurement being performed at a constant room temperature of 25 °C. Maintaining a constant temperature was crucial since the dielectric property of the water-ethanol solutions is influenced by thermal agitation of the dipoles [24,25]. A micropipette was used to precisely introduce 2 µL of each sample to the sensing surface. The samples were iteratively measured three times, and the sensor was cleaned with DI water between successive sample measurements. The measured resonance frequency and the corresponding rejection level of the sensor are summarized in Table S1, which shows that the parameters under consideration did not overlap.

## 3. Results

**Figure 3:**
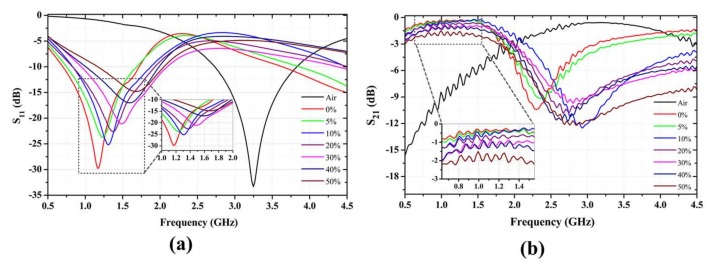
Variation of the peak resonance frequency and the s-parameters (**a**) reflection coefficient (S_11_) and (**b**) transmission coefficient (S_21_) in response to the introduction of various concentrations of the water-ethanol solution.

**Figure 4:**
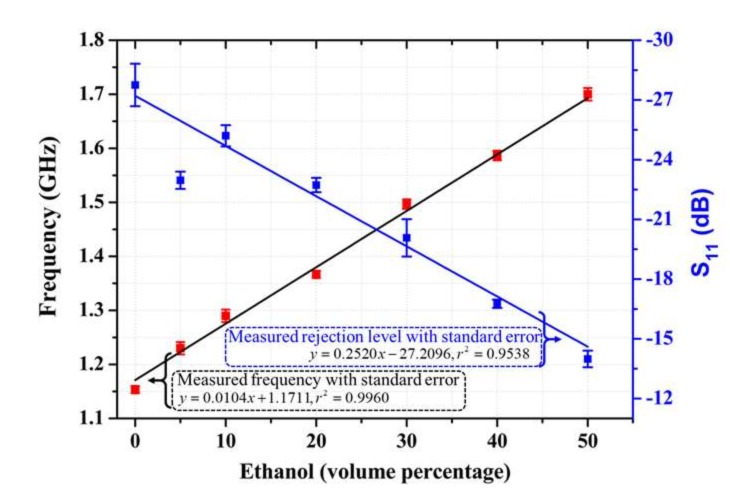
Measurement results of the sensor in terms of the change in frequency and reflection coefficient with respect to the volume percentage of ethanol.

The frequency-dependent response of the water-ethanol solution is shown in Figure 3, which shows increasing resonance frequency with respect to the increase in the volume percentage of ethanol. The resonance frequency increased from 1.16 GHz (for pure DI water) to 1.68 GHz (for 50% ethanol mixture). The resonance peak simultaneously decreased, although a disproportionate value was observed with the 10% ethanol composition. A linear shift occurred in both the resonance frequency and the rejection level, as shown in Figure 4 by the respective positive correlation (*r*^2^ = 0.9938, 0.9560) with the percentage increase of the ethanol. In addition, the respective standard errors of measurement for the resonance frequency and the rejection level did not exceed 0.01 and 1.04 computed for all measurement set (Table S1). The limit of detection (LOD), a measure of the minimum volume percentage of ethanol in the solution that can be effectively detected, was found to be as low as 3.82% for the developed sensor. The increase in resonance frequency and the corresponding decrease in the rejection level were expected because the permittivity of the solution was negatively correlated with the increasing ethanol percentage.

### 3.1. Capacitive Effect

For biosensing applications, the gap capacitance ($C_{g}$) between two resonators is modulated by the external dielectric of the material under test, which in turn varies the open-end admittance of the circuit [26]. The introduction of the aqueous solution to the sensing surface increased the gap capacitance from $C_{g}=C_{u}$, at the unloaded condition to $C_{g}=C_{u}+C_{l}$, at loaded condition. The loaded capacitance ($C_{l}$) is related to the corresponding change in resonance frequency $\left(f_{s}\right)$, which is a function of the solution concentration [27]:

(5) $$C_{l}=\frac{C_{u}\left(f_{o}-f_{s}\right)\left(f_{o}+f_{s}\right)}{f_{s}^{2}}$$

**Figure 5:**
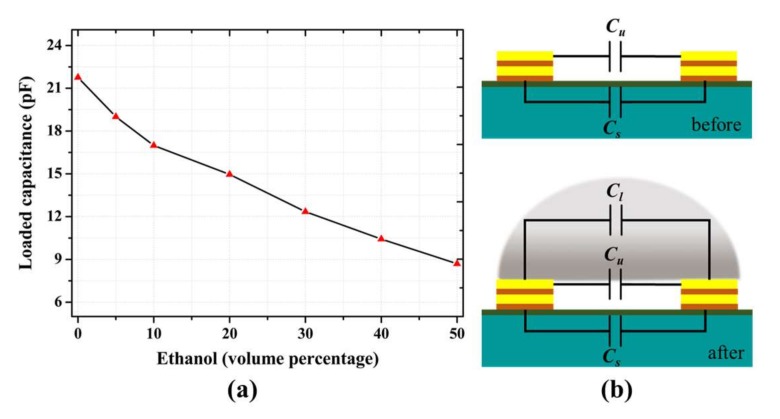
(**a**) Variation in the capacitance as a function of the ethanol concentration. (**b**) Overall capacitance before (top) and after (bottom) the introduction of sample. The introduction of the sample increases the capacitance between the two resonator lines.

The loaded capacitance decreased with the increase in ethanol concentration, as shown in Figure 5. Here, the unloaded capacitance was the value obtained from the simulation result (3.2 pF). The decrease in capacitance was caused by the variation (decrease) of the relative permittivity of the material under test, which is henceforth evaluated in terms of the measurement parameters. The variation in gap capacitance results a linearly fitted sensitivity value of 0.25 pF/ethanol percentage.

### 3.2. Complex Permittivity Analysis

The complex permittivity of different alcohol solutions along with their volume composition with water was documented using the Debye equation [28]. Based on those approximations, we mathematically modeled the relationship between the change in permittivity of the water-ethanol solution and the sensitive radio frequency parameters, namely the resonance frequency $\left(f_{o}\right)$ and the magnitude of the reflection coefficient $\left(\left|S_{11}\right|\right)$, as follows [18]

(6) $$\left[\begin{array}{l} \Delta f_{0}\\ \Delta \left|S_{11}\right| \end{array}\right]=\left[\begin{array}{cc} m_{11} & m_{12}\\ m_{21} & m_{22} \end{array}\right]\left[\begin{array}{l} \Delta {\varepsilon^{\prime }}_{s}\\ \Delta {\varepsilon^{\prime \prime }}_{s} \end{array}\right],$$

where $\Delta \varepsilon =\varepsilon_{s}-\varepsilon_{r}$, $\Delta f_{o}=f_{s}-f_{r}$, $\Delta \left|S_{11}\right|=S_{11s}-S_{11r}$, and subscripts *r* and *s* represent the reference and sample values, respectively. We picked the 30% volume concentration of ethanol as our reference. The unknown coefficients of the matrix were obtained using the least-squares method in order to approximate the complex permittivity of an unknown composition of the solution as follows:

(7) $$\left[\begin{array}{l} \Delta {\varepsilon^{\prime }}_{s}\\ \Delta {\varepsilon^{\prime \prime }}_{s} \end{array}\right]=\left[\begin{array}{cc} -46.4460 & -0.54911\\ -24.2557 & 1.3126 \end{array}\right]\left[\begin{array}{l} \Delta f_{0}\\ \Delta \left|S_{11}\right| \end{array}\right].$$

Thus, the respective complex permittivity values of each sample can be obtained as

(8) $$\begin{array}{l} {\varepsilon^{\prime }}_{s}=\Delta {\varepsilon^{\prime }}_{s}+{\varepsilon^{\prime }}_{r}\\ {\varepsilon^{\prime \prime }}_{s}=\Delta {\varepsilon^{\prime \prime }}_{s}+{\varepsilon^{\prime \prime }}_{r} \end{array}.$$

**Figure 6:**
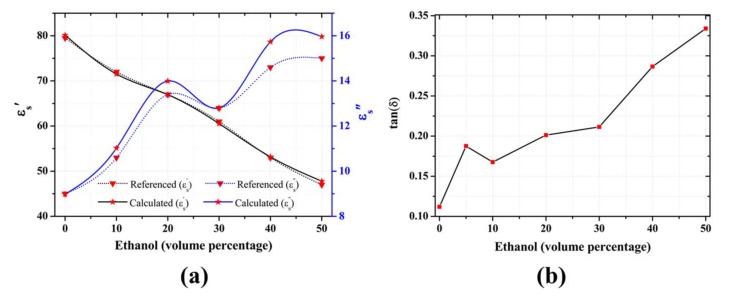
(**a**) Comparison between literature values (triangles) of the real (black) and imaginary (blue) complex permittivity and those obtained from the proposed sensor (stars); (**b**) Increased dissipation factor corresponding to the increase in ethanol concentration.

Figure 6a shows the complex permittivity values of our predictive model, along with those from the literature, and the data seem to be in good agreement, resulting in an average standard deviation of 0.8% and 5.15%, respectively for real and imaginary permittivity Furthermore, the change in the loaded capacitance ($C_{l}$) as a function of concentration of the solution was linearly fitted to the corresponding change in the relative permittivity (${\varepsilon^{\prime }}_{s}$) of the solution with a correlation (*r*^2^) of 0.9758, as shown in supplementary Figure S3, using the following relation:

(9) $$C_{l}=0.40{\varepsilon^{\prime }}_{s}+11.24.$$

From the calculations, we observed that the dissipation factor, i.e., the tangent of the loss angle $\left(\tan(\delta )=\varepsilon^{"}/\varepsilon^{'}\right)$, increased as a function of ethanol concentration, as shown in Figure 6b. The dissipation factor primarily depends on ionic conduction and dipole rotation in the microwave region [29] and has been previously reported to increase with the increase of ethanol in an acid solution [30]. We also speculate that the increase in the dissipation factor explains the decreased reflection coefficient observed at higher concentrations of ethanol.

### 3.3. Reproducibility

The reproducibility of the sensor was verified in two different approaches. First, a repetitive measurement of the samples was performed for three successive iterations, and the response was found to be stable for all measurements, as summarized in Table S1. The fundamental resonance frequency was perfectly regained to 3.24 GHz after the surface was cleaned with DI water and dried.

**Figure 7:**
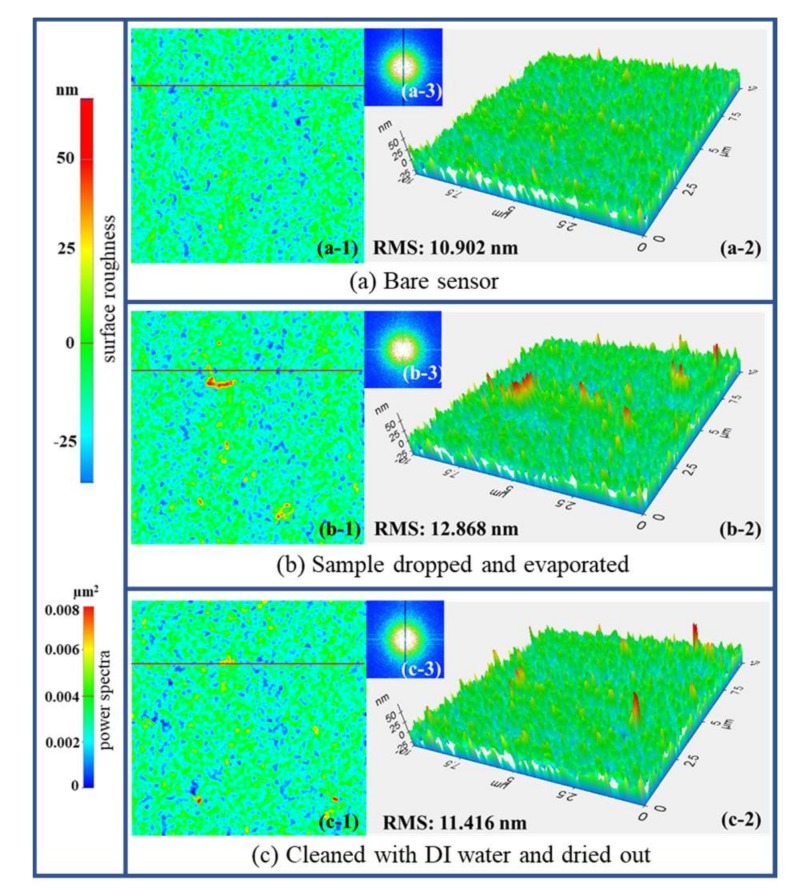
Surface morphological study of the sensor under three sequential conditions. (**a**) Bare chip, (**b**) the water-ethanol solution (containing 30% ethanol) was dropped in the sensing region and allowed to evaporate for 5 min, and (**c**) the surface was cleaned with DI water and then dried. The two-dimensional surface morphology is shown in subfigures (a-1) to (c-1), and the corresponding three-dimensional surface is shown in subfigures (a-2) to (c-2). The power spectra obtained by Fourier transform shown in subfigures (a-3) to (c-3) reveals no significant variation in power at all three conditions.

We further measured the surface morphology of the exposed metal layer to the solution in order to determine the surface quality after repeated measurements. No significant morphological variation was observed in the sensor surface, as shown in Figure 7. Furthermore, we observed that multiple experiments resulted in antifouling of the surface under direct interaction with the solution. The AFM results suggested that no considerable degradation occurred in the surface morphology, thereby maintaining the surface quality of the original device. The RMS values from before and after the test was around 10.902 nm and 11.416 nm, respectively. The morphological study was performed (a) using the surface of the bare sensor, (b) after the sample had been dropped and then exposed to room temperature for 5 min, and (c) after the surface had been cleaned with DI water and dried. Furthermore, the RMS values taken at the same surface line (7.5 µm) were found to be 10.902 nm, 12.868, and 11.416 nm, respectively, for conditions (a)–(c), which revealed that there was no significant variation in the surface morphology. The bumps observed in conditions (b) and (c) could be attributed to nanoparticles of water, which would take a longer time to dry than nanoparticles of ethanol.

The comparison Table 1 is presented below summarizing the performance of our proposed sensor with the similar literatures. Our sensor outperforms the previously reported sensor operating in the similar frequency band with better sensitivity of 10.4 MHz/ethanol percentage and all the cited sensors in terms of selectivity accounting for more than 14 dB rejection.

## 4. Conclusions

In this study, we presented a sensor based on a microfabricated RF resonator by using the passive components of the resonator as vital constituents in the characterization and concentration determination of the water-ethanol solution. Specifically, derived parameters such as the change in gap capacitance and complex permittivity were analyzed based on the variation in the resonance frequency and reflection coefficient as a function of different solution concentrations. The reproducibility of the proposed sensor was verified through iterative sample measurements and surface morphology studies. These findings provide the relevance of the proposed concept as an alternative sensitive detector of the water-ethanol solution at real time.

## Figures and Tables

**Table 1:** Performance comparison of the proposed sensor with the literature.

Reference	Design Concept	Frequency Band of Operation	Sensitivity (MHz/Ethanol Percentage)	Selectivity (in dB)	Reproducibility
**[6]**	Planar resonator	Ku-/K-band	70.72 *	<−8.5 *	NA
**[17]**	Microstrip BPF	<L-band	2.88 *	NA	NA
**[18]**	Split ring resonator	L-/S-band	3.86 *	<−6 *	NA
**This work**	**Spiral-coupled resonator**	**L-/S-band**	**10.4**	**<−14**	**Yes**

*—approximated values, NA—data not available.

## Supplementary Materials

The following materials are available online at http://www.mdpi.com/1424-8220/18/4/1075/s1, Figure S1: Passive microresonator sensor fabrication, Figure S2: Performance of the bare sensor, Figure S3: Loaded capacitance versus relative permittivity, and Table S1: Measured resonance frequencies (in GHz) and corresponding rejection levels (in dB) for three different iterations.
