# Supplementary material for: Radio Frequency Detection and Characterization of Water-Ethanol Solution through Spiral-Coupled Passive Micro-Resonator Sensor
Source: Sensors (Basel). 2018 Apr 3;18(4):1075. doi: 10.3390/s18041075 (PMC5948501; doi:10.3390/s18041075)
Supplement: Supplementary file 1 [file sensors-18-01075-s001.pdf]

sensors

Supplementary Material

Radio Frequency Detection and Characterization of Water-Ethanol Solution through Spiral-Coupled Passive Micro-Resonator Sensor

Gyan Raj Koirala [1], Rajendra Dhakal [1,2], Eun-Seong Kim [1], Zhao Yao [1] and Nam-Young Kim [1,*]

1 RFIC Lab, Department of Electronic Engineering, Kwangwoon University, 01897, Seoul, South Korea; thegrkoirala@gmail.com (G.R.K.); 3037eskim@gmail.com (E.-S.K.); yao9074@hotmail.com (Z.Y.)
2 Department of Computer Science and Engineering, Sejong University, 05006, Seoul, South Korea; rajendra@sejong.ac.kr
* Correspondence: nykim@kw.ac.kr

Received: 9 February 2018; Accepted: 31 March 2018; Published: 3 April 2018

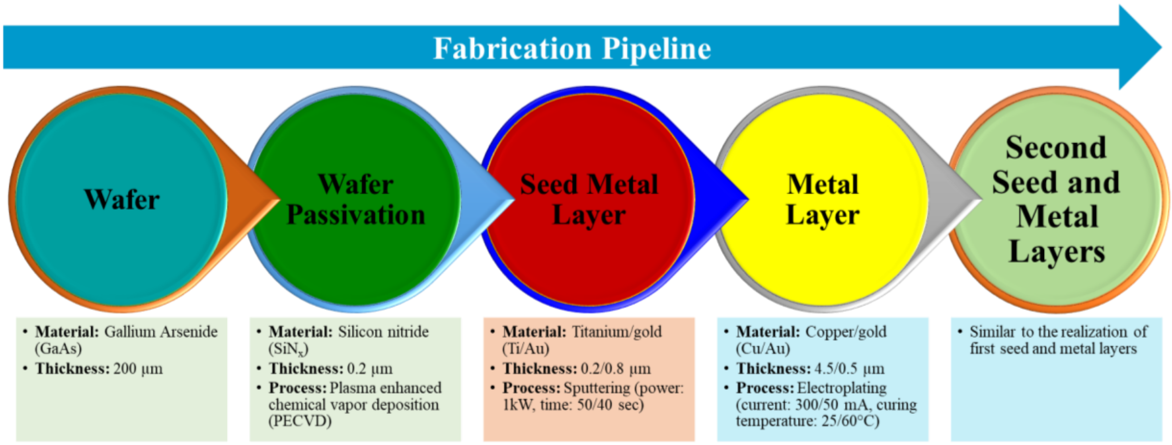

Figure S1. Passive microresonator sensor fabrication. Realization of the passive microresonator sensor using home-developed IPD fabrication processing.

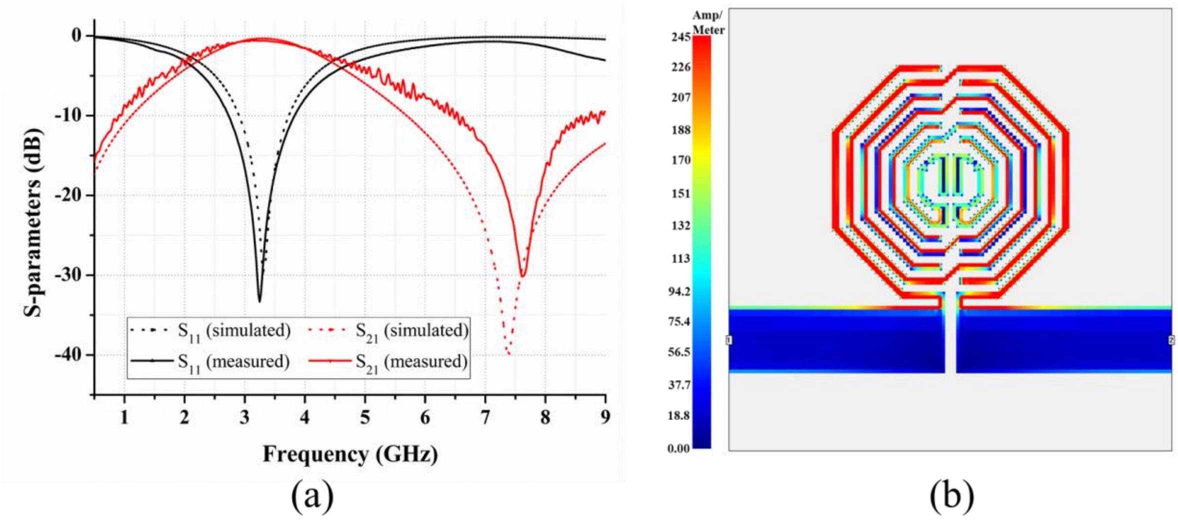

Figure S2. Performance of the bare sensor. (a) Comparison between the simulated and measured scattering parameters, and (b) distribution of the current source density at a simulated resonance frequency of 3.3 GHz.

Table S1. Measured resonance frequencies (in GHz) and corresponding rejection levels (in dB) for three different iterations.

	**Iteration 1**		**Iteration 2**		**Iteration 3**		**Mean ± SE**	
	**RF**	**S_{11}**	**RF**	**S_{11}**	**RF**	**S_{11}**	**RF**	**S_{11}**
DI Water	1.16	-27.44	1.16	-29.74	1.14	-26.08	1.15±0.006	-27.75±1.06
5%	1.21	-22.32	1.23	-23.78	1.25	-22.80	1.23±0.010	-22.96±0.43
10%	1.31	-26.20	1.29	-25.04	1.27	-24.36	1.29±0.010	-25.20±0.53
20%	1.36	-23.40	1.36	-22.62	1.38	-22.16	1.37±0.006	-22.73±0.36
30%	1.50	-20.92	1.48	-21.10	1.51	-18.20	1.50±0.008	-20.07±0.94
40%	1.57	-16.91	1.59	-17.02	1.60	-16.35	1.59±0.008	-16.76±0.20
50%	1.72	-13.70	1.68	-14.81	1.70	-13.46	1.70±0.010	-13.99±0.41

DI: deionized; RF: resonance frequency; Mean ± SE: Mean ± Standard Error

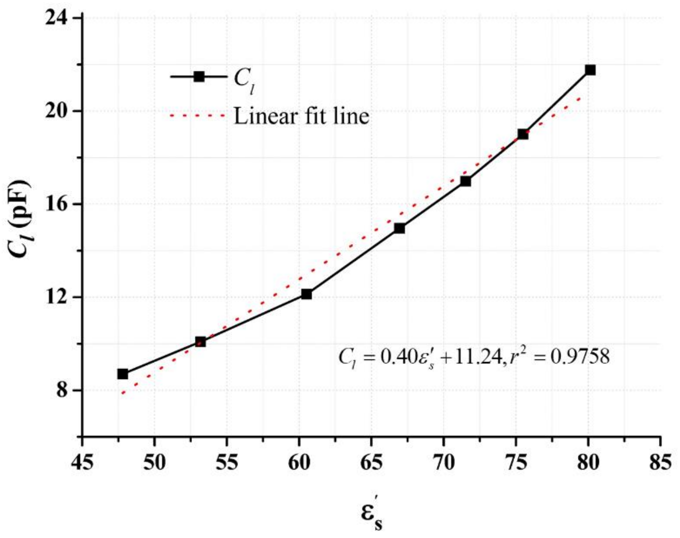

Figure S3. Loaded capacitance versus relative permittivity. Relationship between the computed values of the gap capacitance (C_l) and the relative permittivity (ε_s') of the solution.
